# Moxifloxacin Induced Liver Injury by Causing *Lachnospiraceae* Deficiency and Interfering with Butyric Acid Production through Gut–Liver Axis

**DOI:** 10.1155/2022/9302733

**Published:** 2022-04-29

**Authors:** Yuan Sun, Ling Cong, Song Yang, Rui Zhao, Zhuoling An, Lihong Liu

**Affiliations:** Pharmacy Department of Beijing Chao–Yang Hospital, Capital Medical University, Beijing 100020, China

## Abstract

Cases of unpredictable, idiosyncratic liver damage of moxifloxacin (MXF) have been occasionally reported. However, the health effects of MXF exposure remain controversial. The current study examined the metabolic phenotypes and intestinal flora characteristics of hepatotoxicity induced by MXF. Rats were administered moxifloxacin hydrochloride tablets at doses of 36, 72, and 108 mg/kg body weight/day for 21 days. The levels of tricarboxylic acid cycle intermediates were decreased, whereas those of lipids (arachidonic acid, hexadecanoic acid, and linoleic acid) were increased, reflecting disorders of energy–related and lipid metabolism. Enrichment analysis of the differential metabolites suggested that butanoate metabolism was associated with MXF–induced liver injury. 16S rRNA sequencing uncovered that the diversity of gut intestinal was decreased in MXF–treated rats. Specifically, the abundance of *Muribaculaceae* was increased, whereas that of *Lachnospiraceae*, a family of butyrate–producing bacteria, was decreased. The combined serum metabonomics and gut microbiome datasets illustrated the involvement of butanoic acid and energy metabolism in the regulatory changes of the gut–liver axis associated with MXF–induced liver injury. The regulation of endogenous small molecules and intestinal flora during drug–induced liver injury was first described from the perspective of the gut–liver axis, providing a research basis for the mechanism of clinical drug–induced liver injury.

## 1. Introduction

Liver injury induced by both prescription and over–the–counter drugs is a growing public health problem. Antibiotics represent a relatively frequent cause of acute noninfectious liver injury in the general population [1]. Fluoroquinolones are the most widely prescribed antibiotics because of their high oral bioavailability and broad antimicrobial coverage [2]. Despite their popularity, safety concerns have led to the restriction and even withdrawal of several members of this class of drugs such as trovafloxacin [3].

The application of moxifloxacin (MXF), a fluoroquinolone used to treat respiratory, reproductive tract, and cutaneous infections, has increased in recent years [4, 5]. An American insurance claims study revealed that MXF–induced liver injury occurred at a rate of 16.9/100,000 prescriptions, more than twice as high as amoxicillin–clavulanic acid [6]. The European Medicines Agency and North American regulators issued a warning describing the risk of MXF–associated liver injury and recommended stricter limits on its clinical application [7–10]. A case–controlled study found that the risk of liver injury was higher for MXF and levofloxacin than for other fluoroquinolones [11]. A retrospective study and case report similarly suggested that MXF exposure increased the risk of hepatotoxicity [3, 12]. Although cases of hepatic toxicity induced by MXF have been reported, a comprehensive understanding of the underlying mechanism has not yet been achieved [13].

Metabonomics provides a method platform and basis by which multiple environmental toxicants might interact with a host organism to produce their overall phenotype [14]. Nevertheless, the alterations of endogenous small–molecule metabolites induced by MXF treatment were interpreted diversely because of the limited analysis methods and a lack of validation studies on other omics in scant previous studies. Amino adipic acid, 2–hydroxy–3–methylbutyric acid, and palmitoleic acid were considered the most specific metabolites, whereas Zhou et al. and Leonard et al. found that pyrimidine and purine metabolic pathways were most strongly affected by MXF treatment [15, 16]. Hippuric acid, indole–3–acetate, and glycerol were labeled as pivotal metabolites participating in microbe–associated liver injury in antibiotic–treated rats [17].

Broad–spectrum antibiotics have a profound and long–lasting impact on the composition of the microbiota, including losses of diversity and shifts in community composition [18, 19]. In recent years, a number of studies indicate that changes in the intestinal flora are related to the pathogenesis of numerous diseases, and dietary or environmental factors can affect the composition of the gut microbiota and development of liver diseases, including nonalcoholic steatohepatitis (NASH), nonalcoholic fatty liver disease (NAFLD), and cirrhosis [20–22]. The gut microbiota possibly has a deep-seated effect on host metabolism and health through enterohepatic circulation and the gut–liver axis [23, 24]. Several studies demonstrated that MXF reduced bacterial diversity in a concentration–dependent manner [25, 26]. MXF was less resistant to the colonization of ertapenem–resistant *Escherichia coli* colonization [27]. However, few studies have examined the specific changes of intestinal microflora to reveal the mechanism of MXF–induced liver injury.

Combining metabonomics and other omics provides a promising strategy for obtaining mechanistic insights into various impacts on the microbial community and, in the longer term, on host outcomes. Ishii et al. explored the effects of the American diet on the intestinal environment in mice using an integrated metabonomics and microbiome analysis approach and described positive correlations among increased levels of butyrate, the relative abundance of butyryl–CoA/acetate–CoA transferase, and *Oscillospira* and *Ruminococcus* counts [27]. An integrated omics strategy has been implemented to elucidate the molecular mechanism underpinning the hepatotoxicity of tacrine, and individuals with a higher abundance of *Lactobacillus*, *Bacteroides*, and *Enterobacteriaceae* were more sensitive to tacrine–induced hepatotoxicity, which provided insights for personalized medicine initiatives [24]. The existing studies on metabonomics or intestinal flora were not in–depth research, and the correlations of these factors have not been examined. To date, no study investigated the relationship between metabolic features in the host and the alterations of the gut microbiota in MXF–induced liver injury from the perspective of the gut–liver axis.

We speculated that orally administered MXF might affect the gut–liver axis in rats and thereby induce hepatotoxicity as indicated by metabolic disorders and gut microbe dislocation. In the present study, an integrated metabonomics and intestinal microflora analysis approach were developed for comprehensive metabolite profiles and the structure and abundance of the gut microbiota in rats treated with MXF using liquid chromatography–high resolution mass spectrometry (LC–HRMS) and 16S rRNA sequencing analysis. Our study first verified the potential phenotypes of metabolites and intestinal microbes involved in the gut–liver axis and examined hepatotoxicity induced by moxifloxacin hydrochloride tablets.

## 2. Material and Methods

### 2.1. Chemicals

Analytical–grade methanol (MeOH), acetonitrile (ACN), isopropanol (IPA), and methyl tertiary butyl ether (MTBE) were purchased from Fisher Scientific Co., Ltd (Fair Lawn, NJ, USA). Formic acid (FA) and ammonium acetate (NH_4_OAc) were purchased from Merck (Darmstadt, Germany). Ultrapure water was provided by Wa–Haha Corporation (Hangzhou, China). Moxifloxacin hydrochloride tablets were produced by Bayer Pharma AG (Leverkusen, Germany). Anticleaved caspase–3 antibody was obtained from Abcam (ab2302).

### 2.2. Animal Study and Sample Collection

**Figure 1:**
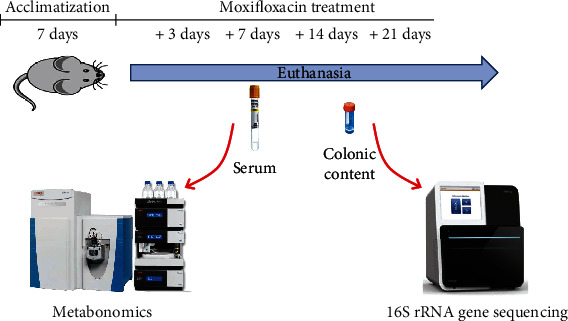
The flow chart of the experimental research framework. Moxifloxacin (0, 36, 72, and 108 mg/kg body weight) was administered orally for 21 days. In each group, six rats were euthanized after anesthetization on days 3, 7, 14, and 21. Serum and colon contents were collected at each time point for metabonomics and 16S rRNA gene sequencing, respectively.

Male Sprague–Dawley rats (*n* = 102, 6–8 weeks old) were purchased from Beijing Vital River Laboratory Animal Technology Co., Ltd. (China). The experimentation on the animal was approved by the Animal Ethics Committee of Beijing Chao–Yang Hospital affiliated with Capital Medical University (China). The animals were maintained in an air–conditioned room at a temperature of 24 ± 2°C, relative humidity of 50 ± 5%, and a 12 h/12 h light/dark cycle. Water and food were available ad libitum during the entire experiment. After 1 week of adaptation to laboratory conditions, the rats were randomly divided into four experimental groups (24 rats/group). Moxifloxacin hydrochloride tablets were prepared as suspensions in distilled water. According to the commonly used clinical dose, suspensions of moxifloxacin (0, 36, 72, and 108 mg/kg body weight) were administered to rats via oral gavage daily. The flow chart of the experimental research framework is presented in Figure 1. Rats in the different dose groups were sacrificed on days 3, 7, 14, and 21. According to the time point and dosage of administration, animals were grouped as follows: low dose, middle dose, high dose, and before administration. Blood was obtained from the abdominal aorta at the indicated time points. Serum was collected via centrifugation at 5000 × *g* and 4°C for 5 min and immediately used for biochemical analysis. Fecal samples were carefully removed from the colon, collected in cryogenic vials, and snap–frozen in liquid nitrogen at once. Then, the serum collected for metabonomics and colonic content collected for 16S rRNA gene sequencing were stored at -80°C. Liver tissues were fixed in 4% paraformaldehyde for pathological examination. The liver tissues were trimmed, embedded in paraffin wax, and then sectioned. The slices were stained with hematoxylin and eosin (H&E) for histopathological examination.

### 2.3. Biochemical Analysis

The analyzed biochemical indices included alanine aminotransferase (ALT), aspartate aminotransferase (AST), alkaline phosphatase (ALP), total bilirubin (TBIL), superoxide dismutase (SOD), and malondialdehyde (MDA). Serum biochemical analyses were performed using a clinical biochemistry analyzer (AU480, Olympus, Japan) at Beijing De–Yi Biotechnology Co., Ltd. (China). Liver oxidative stress biomarkers were examined using visible spectrophotometers (Nanjing Jiancheng Bioengineering Institute, China).

### 2.4. TUNEL Assay

Tissue cell apoptosis was detected using a TUNEL fluorescence assay kit. Liver sections were treated with 0.3% Triton X–100, and after washing, the TUNEL detection solution was added for 1 h. Nuclei were counterstained with DAPI. Antifluorescence quenching sealing agents were added. The samples were visualized by confocal microscopy.

### 2.5. Immunofluorescence

IF–TSA (IF–Tyramide Signal Amplification) staining was used for the simultaneous labeling of proteins. Paraffin–embedded liver tissue was cut to a thickness of 5 *μ*m and dewaxed in xylene and rehydrated in descending concentrations of ethanol. Then, sections were processed for antigen retrieval by incubation in 10 mM sodium citrate buffer (pH = 6.0) containing 0.05% Tween 20 at 95°C for 10 min, washed twice with 0.1% Triton X–100 in PBS, and blocked for 45 min in 3% donkey serum in 0.1% Triton X–100 in PBS. Tissue slices were incubated overnight at 4°C with a primary antibody targeting cleaved caspase–3, followed by incubation with HRP labeled goat anti–rabbit secondary antibody for 1 h and washed three times with PBS. Finally, sections were incubated with CY3–TSA in the dark for 30 min and washed three times with TBST, then bathed in antigen repair solution (pH = 8.0) in a microwave to remove the primary and secondary antibodies bound to the tissue. After immunostaining, sections were counterstained with 5 *μ*g/mL DAPI for 10 min in the dark at room temperature and mounted with antifade mounting medium (Beyotime, Jiangsu, China). HistoQuest image analysis software was used to differentiate cleaved caspase–3 and DAPI–positive cells. Tissue virtual microscopy analysis was then performed using the soft system.

### 2.6. Serum Sample Preparation

To each 50 *μ*L aliquot of serum, 750 *μ*L of MeOH and 750 *μ*L of MTBE were added to precipitate proteins and extract the metabolites. The mixture was vortexed for 5 min and then centrifuged at 15,000 rpm (4°C, 10 min). The supernatant was transferred and added to 600 *μ*L of H_2_O and 750 *μ*L of MTBE. The upper organic portion and lower aqueous portion were separated via vortexing and centrifuged at 15,000 rpm (4°C, 10 min). The polar and nonpolar metabolites were derived from the aqueous and organic phases, respectively. The supernatant was transferred and evaporated to dryness. The polar and nonpolar residues were redissolved in 50 *μ*L of ACN/H_2_O (2 : 98, *v*/*v*) and 50 *μ*L of MeOH/CHCl_3_ (1 : 1, *v*/*v*), respectively, vortexed for 5 min, and then centrifuged at 15,000 rpm (4°C, 10 min). The supernatant was collected for further analysis.

### 2.7. LC–HRMS Analysis of Serum Metabolites

Untargeted metabonomic analysis relying on a Dionex UltiMate 3000 HPLC system (Dionex, Olten, Switzerland) coupled with a Q–Orbitrap mass spectrometer (Q Exactive; Thermo Fisher Scientific, Waltham, MA, USA) via a heated electrospray source. Chromatographic separation was performed on a reversed–phase ACQUITY UPLC HSS T_3_ column (2.1 × 100 mm × 1.7 *μ*m; Waters, Milford, MA, USA), and the temperature was set at 40°C. The mobile phases for polar metabolite detection consisted of 0.1% FA in water (mobile phase A) and ACN (mobile phase B). The mobile phases for nonpolar metabolite detection consisted of 0.1% FA and 2 mmol NH_4_OAc in water (mobile phase A) and 0.1% FA and 2 mmol NH_4_OAc in ACN/IPA (1 : 1, *v*/*v*, mobile phase B). The flow rate and autosampler were set at 0.25 mL/min and 4°C, respectively. The injection volumes of the positive and negative electrospray ionization (ESI) modes were set as 5 *μ*L. The gradient conditions were as follows: 0–3 min, linear gradient of 2%–20% B; 3–10 min, linear gradient of 60% B; 10–15 min, 60%–100% B; and 15–20 min, 100% B. The column was equilibrated for 8 min before injection.

The detailed parameters for MS were as follows: scan mode, full scan; resolution, 70,000; scan range, *m/z* 66.6–1000.0; spray voltage, 3.5 kV; capillary temperature, 350°C; auxiliary gas heater temperature, 220°C; sheath gas flow rate, 40 psi; auxiliary gas flow rate, 11 Arb; and sweep gas flow rate, 0. Atomized gas and collision gas used for high–energy collision dissociation is high–purity nitrogen (N_2_). The data–dependent MS/MS (dd–MS^2^) method was followed by full–scan MS. The operational parameters for targeted MS/MS were as follows: resolution, 17,500; scan range, *m/z* 66.6–1000.0; automatic gain control target, 1e^−6^; max injection time, 100 ms; and isolation window, 3 m*/z*. The collision energies were set at 15, 30, and 45 eV.

### 2.8. Data Processing and Statistical Analysis of Serum Metabolites

Raw LC–HRMS data were converted to the *m/z* format using Mass Matrix MS Data File Conversion software (http://www.massmatrix.net). Then, using XCMS software, peak recognition, filtering, alignment, and scaling were conducted, and a two–dimensional data matrix including *m/z*, the retention time, and the peak area was acquired. For polar extracts, Mummichog [28, 29] was employed for the standardized data for nontargeted metabolite labeling and metabolic pathway analysis using the MetaboAnalyst website (http://www.metaboanalyst.ca/). MATLAB was further used to analyze the ratio of directly related metabolites in the metabolic network generated by the aggregation of metabolic pathways for which gamma *p* < 0.05. For nonpolar extracts, mass spectra data were imported into SIMCA–P for orthogonal partial least squares discriminant analysis (OPLS–DA) analysis and intergroup differential metabolite screening (VIP > 1.5, *p* < 0.05, and delete Jack–Knifed over zero value). The metabolites obtained after screening were identified using the Lipidmaps database (https://www.lipidmaps.org/) according to the precise mass number.

### 2.9. 16S rRNA Gene Sequencing and Analysis

Genomic DNA was extracted from colonic content using a QIAamp 96 PowerFecal QIAcube HT Kit (Qiagen, Hilden, Germany) in accordance with the operation instruction manual. All operations were performed in a sterile environment. The DNA concentration was checked using a NanoDrop spectrophotometer (Thermo Scientific), and DNA quality was tested using 1% agarose gel electrophoresis. The 16S rRNA gene hypervariable V3–V4 region was amplified by PCR for 25 cycles using 1 *μ*L of template DNA (10–50 ng/*μ*L), 12.5 *μ*L of KAPA HiFiHotStart Ready Mix (Anachem, Dublin, Ireland), and 25 *μ*M of each primer (Bakt_343F, 5′–TACGGRAGGCAGCAG–3′; and Bakt_798R,5′–AGGGTATCTAATCCT–3′) in a 25 *μ*L reaction volume. The first PCR program setup consisted of the following steps: 3 min at 95°C; 25 cycles of 30 s at 95°C, 30 s at 55°C, and 72°C for 30 s; and then 5 min at 72°C. The conditions for the second PCR were the same as that of the first PCR, and the sequencing adaptors and primers were attached to the amplicon library with eight cycles. Purification of the amplicon products was performed using of Agencourt AMPure XP Beads (Beckman Coulter Genomics, MA, USA). The amplicon products were quantified by densitometry using Quantity One software (Bio–Rad, Hercules, CA, USA) and mixed in equimolar amounts. The DNA library was purified by gel extraction using a Qiagen gel extraction kit. The concentration and length distribution of the DNA library were checked using a Qubit Fluorometer (Invitrogen, Carlsbad, CA, USA) and Qseq100 (BiOptic Inc., Taiwan, China). The V4 region of bacterial 16S rRNA genes was sequenced using the Miseq PE300 platform as mentioned above. The aforementioned experimental operations were performed by Shanghai OE Biotechnology Co., Ltd. (China).

Raw sequencing data were in FASTQ format. Paired–end reads were then preprocessed using Trimmomatic software to detect and remove ambiguous bases (N) [30]. The software also removed low–quality sequences with an average quality score of less than 20 using a sliding window trimming approach. After trimming, paired–end reads were assembled using FLASH software [31]. The parameters of assembly were as follows: minimal overlap of 10 bp, maximal overlap of 200 bp, and maximal mismatch rate of 20%. Sequences were subjected to further denoising as follows: reads with ambiguous, homologous sequences or sequences less than 200 bp in length were abandoned, reads with 75% of bases above Q20 were retained, and then reads with chimeras were detected and removed. These steps were performed using QIIME software (version 1.8.0) [32]. Clean reads were subjected to primer sequence removal and clustering to generate operational taxonomic units (OTUs) using Vsearch software with a 97% similarity cutoff [33]. The representative read of each OTU was selected using the QIIME package. All representative reads were annotated and blasted against the Silva database Version 132 (or Greengens) (16S rDNA) using the RDP classifier (confidence threshold was 70%) [34].

## 3. Results

### 3.1. Hepatic Injury Induced by Moxifloxacin Hydrochloride Tablets

**Figure 2:**
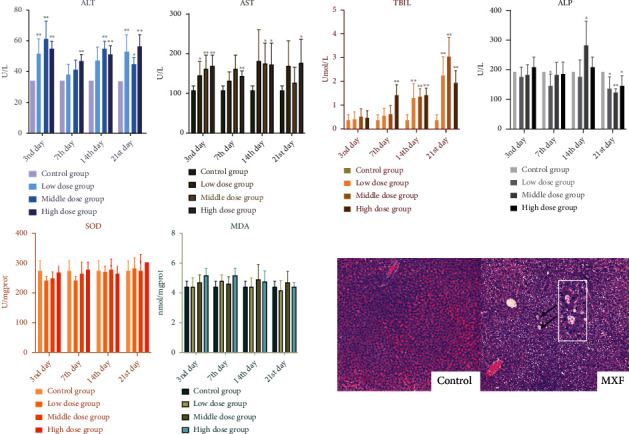
Hepatic injury was appraised using liver injury correlative serum biochemical indicators and histopathological observation. The serum levels of alanine aminotransferase (ALT), aspartate aminotransferase (AST), total bilirubin (TBIL), alkaline phosphatase (ALP), superoxide dismutase (SOD), and malondialdehyde (MDA) levels in rats were detected on days 3, 7, 14, and 21 of moxifloxacin (MXF) treatment (^∗^*p* < 0.05). Representative pathological images of hematoxylin and eosin (H&E) staining under light microscopy revealed fatty degeneration in hepatic cells, which presented as fat vacuoles (black arrow), in MXF–treated rats (magnification: 10x).

The changes in transaminase levels and the histopathology of hepatic injury induced by MXF are presented in Figure 2. Excluding MDA and SOD, there were varying degrees of conspicuous changes of other biochemical indices. Compared with the findings in the corresponding control groups, AST and ALT levels were significantly increased by different MXF doses on days 3, 7, 14, and 21. In addition, TBIL levels tended to increase over time, and ALP levels were significantly different between the control and treatment groups on day 14. Meanwhile, pathological damage in liver tissue sections was also identified on day 7 in the high–dose group. Fatty degeneration of hepatocytes, which was denoted by the presence of fat vacuoles, was evident in MXF–treated rats.

**Figure 3:**
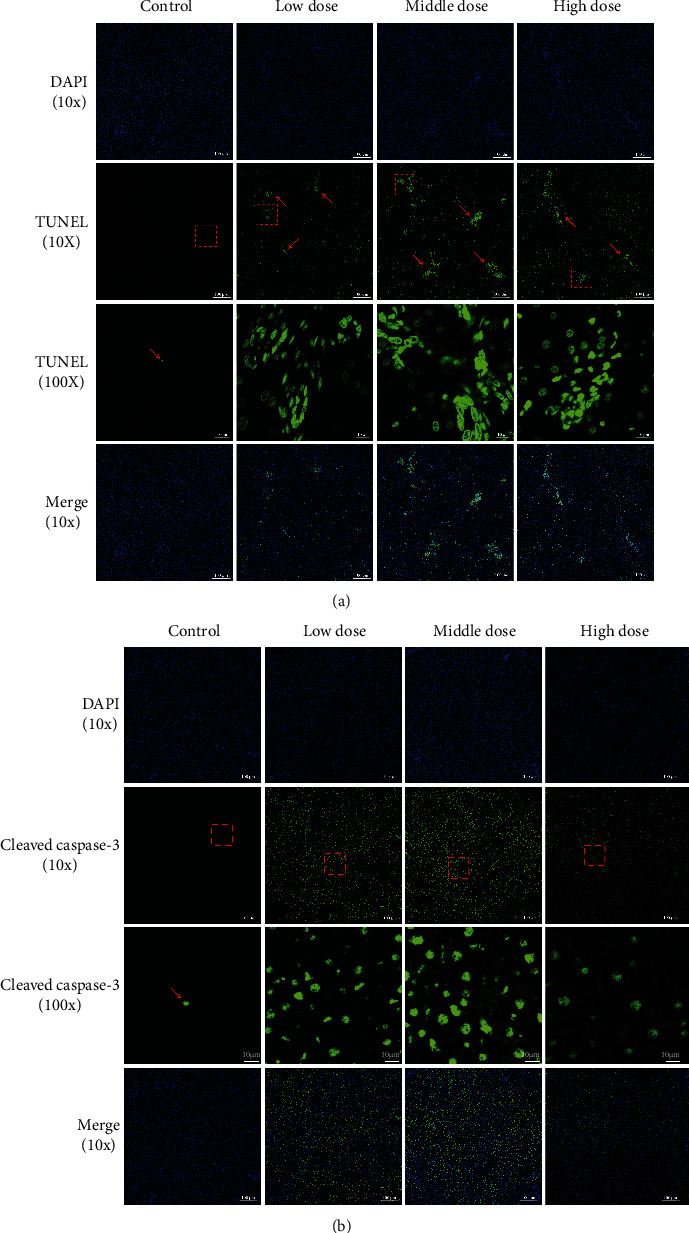
TUNEL analysis and immunofluorescence of the expression of cleaved caspase–3 in rats' liver tissues with moxifloxacin- (MXF-) induced liver injury on day 7 of treatment, when liver damage was most severe. Liver tissues were stained with TUNEL reaction to reveal cells undergoing DNA fragmentation (a). Sections of liver tissues were processed for TUNEL assay to detect apoptosis of the liver. Nuclei were stained with DAPI (blue), and the section undergoing TUNEL assay was stained in green. Magnification: 100x. The expression of cleaved caspase–3 in liver tissues was observed by a fluorescence microscope (b). Cleaved caspase–3 (green) and DAPI staining (blue), as well as merged pictures, are presented. Magnification: 100x.

Furthermore, we performed TUNEL analysis to evaluate the apoptosis in vivo (Figure 3(a)) and immunofluorescence to observe the expression of cleaved caspase–3, a cell death marker in liver tissue (Figure 3(b)). All different doses of moxifloxacin administration resulted in the apoptosis of hepatocytes. According to the results of liver enzymology, the degree of liver injury appeared to be independent of the dose. The results in TUNEL analysis and immunofluorescence similarly did not find a correlation between the degree of damage and the dose level, which conformed to the definition of idiosyncratic drug–induced liver injury.

### 3.2. Metabonomic Profiles and Metabolic Characteristics

**Figure 4:**
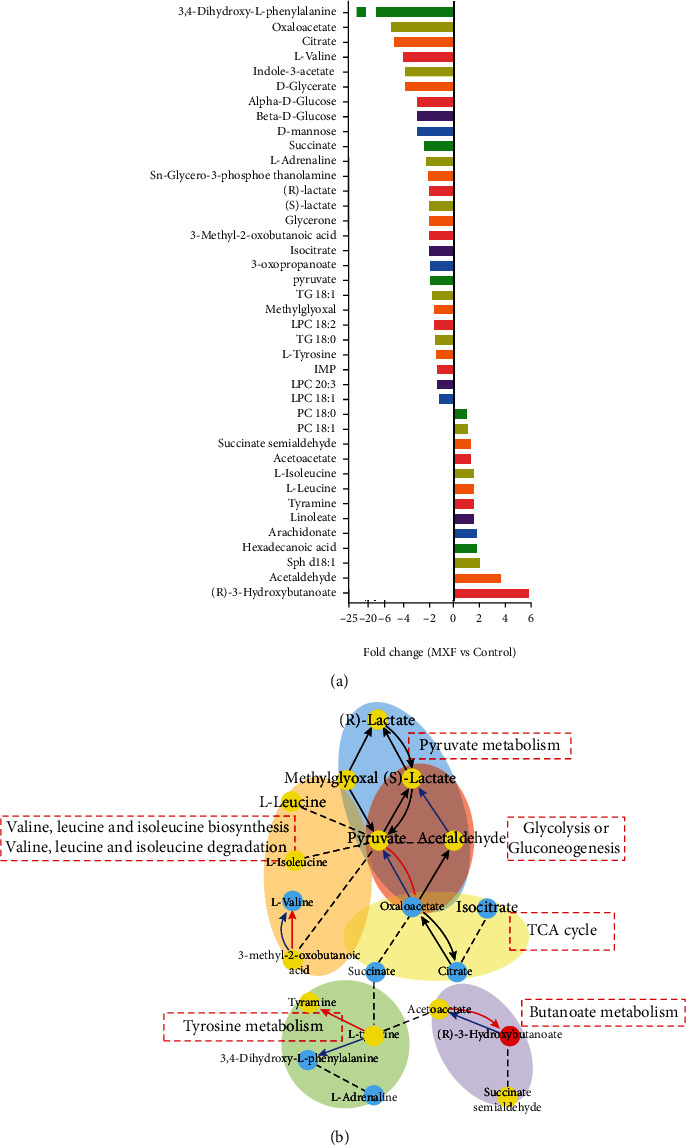
Metabonomic analysis and metabolic changes in rats with moxifloxacin- (MXF-) induced liver injury on day 7 of treatment, when liver damage was most severe. (a) The fold changes of 40 polar and nonpolar metabolites were identified. FC > 0, upregulated metabolites; FC < 0, downregulated metabolites. (b) The metabolites with mutual reaction relationships were visualized, and these relationships involved seven metabolic pathways. The red nodes denote the upregulation of metabolite abundance, the blue nodes denote the downregulation of metabolite abundance, and the yellow nodes represent metabolites with no significant changes. Red edges represent the enzymes that have been activated for two metabolites. Blue edges represent the enzymes that have been inactivated for two metabolites. Black edges represent the metabolite ratios have direct relationships but no significant changes. Metabolites with no direct relationships were linked by dotted lines.

To screen for unique metabolites, we compared the metabolism of control and MXF–treated rats on day 7 when the hepatic damage was most pronounced. Using “the metabolome big data processing method for metabolite labeling and metabolic pathway mapping based on the Mummichog algorithm,” thirty–one unique marker metabolites (*p* < 0.05) and seven relevant metabolic pathways were revealed from polar extract data. Using the SMICA–P software combined with the Lipidmaps database, we uncovered nine lipid metabolites from the nonpolar extract data with significantly different levels between the control and MXF groups (*p* < 0.05) that were associated with MXF–induced liver injury (Figure 4(a)).

**Figure 5:**
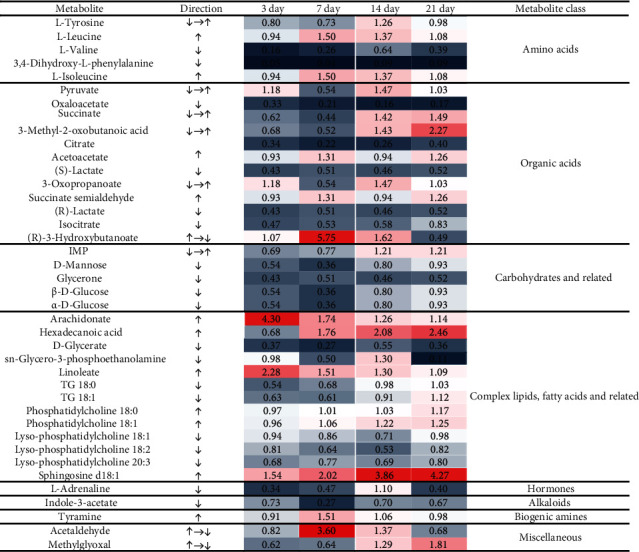
Heatmap of metabolites with significant changes in abundance after 3, 7, 14, and 21 days of moxifloxacin treatment (*p* < 0.05). Red boxes indicate significant increases in abundance relative to the control level, and blue boxes denote significant decreases.

As revealed in Figure 4(b), three of the significant pathways were involved in energy metabolism, including glycolysis or gluconeogenesis, the tricarboxylic acid (TCA) cycle, and pyruvate metabolism. In addition, butanoate metabolism and three other amino acid–related pathways (tyrosine metabolism; valine, leucine, and isoleucine degradation; and valine, leucine, and isoleucine biosynthesis) were also marked. The levels of the aforementioned 40 metabolites on days 3, 7, 14, and 21 according to metabonomic analysis are presented in Figure 5. Compared with the findings in the control group, the levels of 10 metabolites were significantly increased and those of 21 metabolites were significantly decreased by MXF exposure (*p* < 0.05). In addition, the levels of two metabolites increased first and then decreased, and those of four metabolites decreased and then increased. The level of each metabolite in the treatment groups was compared with that in the control group and expressed as a fold change. Changes were observed in the levels of amino acids, organic acids, carbohydrates, complex lipids, fatty acids and related compounds, hormones, alkaloids, biogenic amines, and miscellaneous compounds. The levels of L–valine and 3,4–dihydroxy–L–phenylalanine (levodopa) were decreased at all time points. The abundance of 3–methyl–2–oxobutanoic acid increased gradually over time, and that of 3–hydroxybutyric acid sharply decreased after 7 days of administration. The levels of carbohydrates and related compounds, including inosinic acid, D–mannose, glycerone, and glucose, declined by varying degrees at most time points. Among complex lipids and fatty acids, arachidonic acid, hexadecanoic acid, and linoleic acid levels were changed by 2–fold following MXF exposure for different times. The relative abundance of sn–glycero–3–phosphoethanolamine decreased by 9–fold in the MXF group compared with the control level, but substantial variation was noted among the four–time points. Acetaldehyde and methylglyoxal levels also increased over time.

### 3.3. Changes of the Gut Microbiota

**Figure 6:**
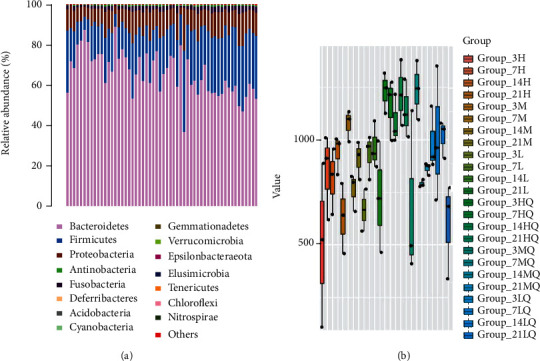
Community structure and *α*–diversity of the gut microbiota following moxifloxacin administration in rats. (a) Fifteen most abundant bacteria at the phylum level. (b) Comparison of the *α*–diversity of the gut microbiota between different groups using diversity index box plots. The abscissa represents groups, with different groups distinguished by different colors, and the ordinate represents index values. L: low dose; M: middle dose; H: high dose; and Q: before administration. The number represents the day of collection (3, 7, 14, and 21 days of treatment).

The composition and structure of the gut microbiota at the phylum level (top 15) are presented in Figure 6. The four most abundant bacteria at the phylum level were *Bacteroidetes*, *Firmicutes*, *Proteobacteria*, and *Actinobacteria* as indicated by *α*–diversity, a measure of within–sample diversity that is calculated using the richness and evenness of bacterial taxa within a single population. The diversity indices of MXF–treated rats were significantly lower than those of control rats, which indicated that the species richness of the intestinal flora in rats was dropped off by MXF administration. The *β*–diversity, which refers to between–sample diversity, measures the distance between pairs of samples. Nonmetric multidimensional scaling (NMDS) analysis based on OUT information was performed to demonstrate the repeatability of each group (stress = 0.094). On days 7 and 14 of administration, the consistency of each dose group was good, and the differences between different groups were marked.

**Figure 7:**
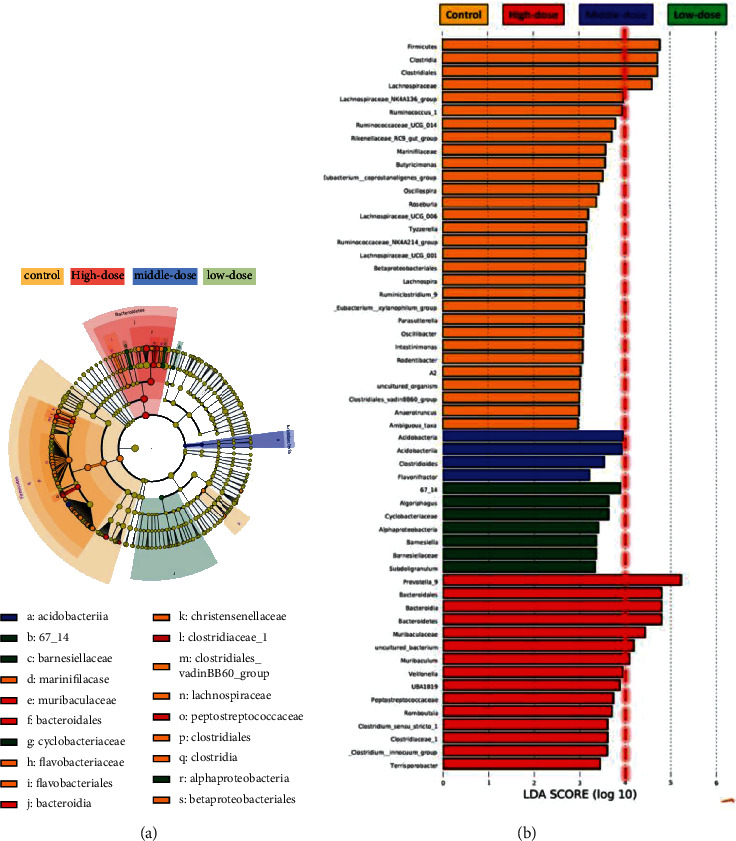
Gut microbiota with differences in abundance between the control and moxifloxacin groups. (a) Evolutionary branch diagram of gut microbiota with differences in abundance. The red nodes imply species with relatively high abundance in the high–dose group, the blue nodes imply species with relatively high abundance in the middle–dose group, and the green nodes imply species with relatively high abundance in the low–dose group. The diameter of nodes is in direct proportion to the relative abundance. The nodes of each layer, from inside out, represent phylum, class, order, family, and genus. (b) Histogram of gut microbes with differences in abundance. Red, blue, green, and yellow bars represent microbes with differences in abundance in the high–dose, middle–dose, low–dose, and control groups, respectively.

Linear discriminate analysis (LDA) coupled with effect size measurements was used to identify strains with differences in abundance among the groups. The branching evolutionary map with the different strains labeled and the primitive data histogram of the relative abundance of different strains are presented in Figure 7. Ten strains exhibited differences in abundance at different taxonomic levels (LDA score > 4) in the aggregate. Subsequently, only three different strains belonging to *Bacteroidetes* and *Firmicutes* were retained after incorporating and retaining the lowest taxonomic level of duplicated results. They were *Firmicutes*–*Clostridia*–*Clostridiales*–*Lachnospiraceae*, *Bacteroidetes*–*Bacteroidia*–*Bacteroidales*–*Muribaculaceae*–*Muribaculum*, and *Bacteroidetes*–*Bacteroidia*–*Bacteroidales*–*Prevotellaceae*–*Prevotella_9*. Compared with the control findings, the abundance of *Lachnospiraceae* was significantly decreased, whereas that of *Muribaculaceae* was significantly increased.

## 4. Discussion

The gut–liver axis is the focus of drug–induced liver injury. The gut microbiome in chemical–induced hepatotoxicity is well established based on pharmacological studies utilizing a variety of chemical–induced metabolic and toxicological disease models [35]. The gut microbiota serves as the key upstream modulator for the progression of chronic liver diseases, including viral hepatitis, fatty liver disease, autoimmune hepatitis, cirrhosis, and liver cancer [36].

MXF, as a broad–spectrum antimicrobial drug, has been reported to alter the growth and composition of gut flora. However, whether MXF–induced liver injury is mediated by the metabolism of endogenous metabolites and the intestinal flora in the gut–liver axis has not been studied. The current experiment thus explored the potential small molecules and intestinal microbes affecting hepatotoxicity in the gut–liver axis of MXF–treated rats. Based on changes in biochemical and pathological indices, including AST, ALT, and TBIL, a rat model of MXF–induced liver injury was successfully established, and biological samples were collected. MXF–induced liver injury in this study appeared to be independent of the dosage, and the changes of pathology and serum transaminase levels within 7 days were consistent with rules of idiosyncratic drug–induced liver injury in earlier reports [13].

LC–HRMS combined with a multivariate data analysis method including Mummichog and OPLS–DA analysis displayed the advantages of network analysis for pathway enrichment, the prediction of functional activity, and putative metabolite identification. Forty unique marker metabolites (*p* < 0.05) and seven relevant metabolic pathways were revealed from polar and nonpolar extract data. The serum levels of metabolites involved in the TCA cycle and oxidative phosphorylation including citrate, oxaloacetate, succinate, and isocitrate were altered by MXF treatment. Oxaloacetate is a key rate–limiting substrate affecting the speed of the TCA cycle. Previous research revealed that ATP production and respiratory fluxes were enhanced after oxaloacetate treatment in SHSY5Y neuronal cells [37], and another related study reported that oxaloacetate promotes brain mitochondrial biogenesis [38]. In addition, a study by Cao et al. on the hepatotoxicity of antituberculosis drugs found that isocitrate levels were significantly decreased in the test group, and networks analysis revealed a link between isocitrate and lipid peroxidation–related compounds [39]. Oxaloacetate and isocitrate levels gradually decreased over time during MXF administration, suggesting that the function of liver mitochondria and adenosine triphosphate (ATP) production might have been impaired. The levels of 3–hydroxybutyric acid, as one of the ketones, rose sharply during treatment, which was also validated. Glucose is completely oxidized into water and carbon dioxide through oxidative phosphorylation. Under abnormal circumstances, glucose is degraded to pyruvate, and a limited amount of ATP is produced via this process [40]. A dynamic balance exists between glycolysis and aerobic oxidation of glucose, and organisms will increase glycolysis to produce ATP when mitochondria are injured [41]. Glycolysis originates the highly reactive toxic byproduct methylglyoxal. Under normal physiological conditions, only a small amount of glucose can be converted into methylglyoxal. Existing studies suggested its involvement in insulin resistance and *β*–cell dysfunction, creating a vicious cycle between glycation and hyperglycemia [42]. In the current study, glycolysis or gluconeogenesis and pyruvate pathways were mapped in the test group, in addition to the aberrant elevation of ketone and methylglyoxal levels, which confirmed that disordered oxidative phosphorylation and glycolysis seem to be characteristic of liver injury induced by MXF.

In addition to the abnormal changes of oxidative phosphorylation and glycolysis, the levels of some endogenous lipids including arachidonic acid, linoleic acid, and hexadecanoic acid were also elevated. Arachidonic acid is a polyunsaturated and essential fatty acid that is synthesized from linoleic acid. It mediates inflammation either directly or indirectly following its conversion into eicosanoids, including prostaglandins, thromboxanes, and leukotriene [43]. Generally, the eicosanoids derived from arachidonic acid are proinflammatory. The significantly increased arachidonic acid concentrations in MXF–treated rats induced a proinflammatory response, which might be related to the hepatotoxicity of MXF. Linoleic acid is the substrate of arachidonic acid, and increased linoleic acid levels have been described as a cause of liver lipid metabolism disorder [44]. Hexadecanoic acid is the main saturated fatty acid that naturally occurs in animal fats, and it has been widely reported in many cell models characterized by cell dysfunction or cell death [45, 46]. In the presence of hexadecanoic acid, the mitochondrial membrane potential of liver cells collapses, causing endoplasmic reticulum stress and liver cell apoptosis. We observed obvious disturbances of energy metabolism relevant metabolites in the present study. Regulatory changes in energy metabolism are considered critical to the pathological changes of steatosis in hepatic cell hepatocytes in MXF–treated rats. Meanwhile, oxidative phosphorylation, glycolysis, and liver lipid metabolism dysfunction reflect the toxic effects of MXF.

According to the comprehensive metabolic analysis, the biosynthesis and degradation of valine, leucine, and isoleucine were seriously affected by MXF administration. Abounding enzymes involved in amino acid metabolism are stored in the liver. Isoleucine and valine are standard amino acids with aliphatic side chains, and thus, they are known as branched–chain amino acids (BCAAs). In this study, we found that serum valine levels were significantly decreased and isoleucine levels were increased by MXF treatment. These findings implied that MXF administration might hamper the utilization of circulating BCAAs. Previous studies reported the conclusive effects of BCAAs in the treatment of patients with liver diseases, and BCAA levels can be used to diagnose liver dysfunction [47, 48]. Interestingly, levodopa exhibited the most dramatic change (9–fold reduction) among all unique metabolites. Levodopa is the precursor of noradrenaline and dopamine in humans, and it serves as an irreplaceable modality for the symptomatic management of Parkinson's disease [49]. To our knowledge, no link between levodopa and hepatotoxicity has been reported. Thus, whether levodopa can be used as a potential biomarker for MXF–induced liver injury requires further verification.

**Figure 8:**
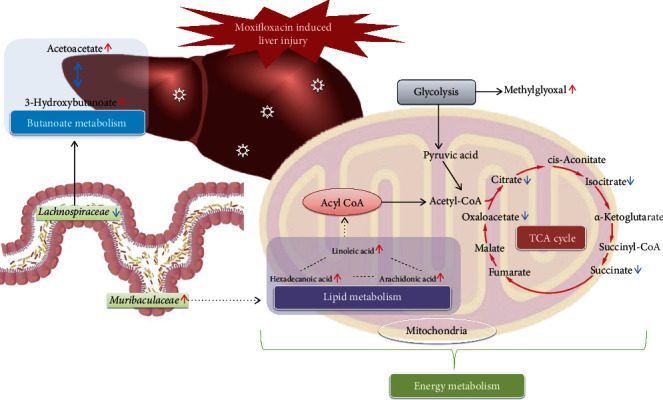
Interaction network of metabonomic profiles, the gut microbiota, and the indirect pathway of the gut–liver axis in moxifloxacin–induced hepatotoxicity. Oxidative phosphorylation, lipid metabolism, and butanoate metabolism are the main metabolic pathways affected by moxifloxacin. The variations of gut flora, represented by a heightened abundance of *Muribaculaceae* and the decline abundance of *Lachnospiraceae*, led to the observed changes in intestinal metabolic function. *Muribaculaceae* and *Lachnospiraceae* participate in the reduction of butyric acid production and alteration in lipid metabolism could be closely related to the pathophysiological changes of liver function.

16S rRNA gene sequencing was performed to profile the composition of the gut microbiota of rats before and after MXF dosing. The results indicated that the structure and composition of the intestinal flora were significantly influenced by MXF. When liver damage was most severe, *Muribaculaceae* counts were significantly increased, and those of *Lachnospiraceae* were significantly decreased. It has been reported that the abundance of *Muribaculaceae* consistently positively covaries with the inner mucus layer (IML) barrier function [50], and the IML is a critical barrier that protects the colonic epithelium from luminal threats and inflammatory factors. Another study identified an increase in the abundance of *Muribaculaceae* in association with resistance to fat [51]. Therefore, *Muribaculaceae* may be related to the immune function of the intestinal mucosa and the absorption and utilization of fat. However, little is known about the relationships between *Muribaculaceae* and liver diseases at present. *Lachnospiraceae* is a member of the phylum *Firmicutes*, and it is linked to short–chain fatty acids (SCFA) production [52]. The decline in the diversity of gut microbiota, especially those involved in the generation of protective SCFAs, indicates this physiological change may be closely related to the pathogenesis of drug–induced liver injury [53]. The conservation of the intestinal barrier is related to the role of SCFAs, including butyrate acid [54, 55]. Butyrate acid can also suppress inflammation and mild liver mitochondrial oxidative stress to reduce the occurrence and development of drug–induced liver injury [56, 57]. Consistent with previous findings, butanoate metabolism was mapped in the disrupted pathway in the metabonomic analysis. Consequently, we proposed that MXF decreased the production of SCFAs including butyrate acid by reducing the abundance of *Lachnospiraceae*, leading to the impaired intestinal mucosal mechanical barrier and immune barrier function (Figure 8). A large number of harmful substances including lipopolysaccharide (LPS) as a representative endotoxin and proinflammatory factor might be released into the blood and lead to toxic effects on the liver because of the increased intestinal permeability. The alteration of the gut–liver axis and cometabolism between the gut microbiota and host liver might be the main mechanism of hepatotoxicity induced by MXF.

## 5. Conclusion

To the best of our knowledge, this is the first study to report metabolic alternations and microbiota composition differences in a model of MXF–induced hepatotoxicity. We proposed the use of metabonomics combined with intestinal microflora analysis of the gut–liver axis to investigate MXF–induced liver injury. MXF induced dose–independent hepatotoxicity after oral exposure. The energy–related metabolic disorders caused by butyric acid deficiency and intestinal microecological disorders are primarily responsible for liver damage based on the changes in serum metabolite levels and intestinal bacterial species abundance. The underlying relationship of the gut–liver axis associated with the gut flora and liver metabolism may act as a critical part of the underlying pathogenesis. Our results provide new insights into the pathogenesis of hepatotoxicity caused by MXF and improve our knowledge of the drug–induced liver injury.

## Data Availability

All research data obtained during the research are open to the industry. If necessary, please contact the corresponding author Professor Lihong Liu (liulihong@bjcyh.com) or the first author Dr. Yuan Sun (sunny5106@163.com) by email. We will get in touch with you within a week.
